# Association of anti-HCV sero-prevalence with blood transfusion and practice of haemodialysis from multiple centres in patients on maintenance haemodialysis

**DOI:** 10.12669/pjms.36.2.1343

**Published:** 2020

**Authors:** Umbreen Amjad, Saqib Qayyum Ahmad, Saima Mir, Moazam Ayub

**Affiliations:** 1 Dr. Umbreen Amjad, MBBS. Department of Pathology, Bahria International Hospital, Rawalpindi, Pakistan; 2 Dr. Saqib Qayyum Ahmad, FCPS. Department of Pathology, Bahria International Hospital, Rawalpindi, Pakistan; 3 Dr. Saima Mir, MD (Nephrology). Department of Nephrology, Bahria International Hospital, Rawalpindi, Pakistan; 4 Dr. Moazam Ayub, MD (Nephrology). Department of Nephrology, Bahria International Hospital, Rawalpindi, Pakistan

**Keywords:** Anti-HCV, Blood transfusion, End stage kidney disease, Maintenance haemodialysis

## Abstract

**Objectives:**

To determine the frequency of anti-HCV in patients on maintenance haemodialysis (HD) and its association with history of blood transfusion and with the practice of HD from more than one center.

**Methods:**

All the patients on maintenance HD at Bahria International hospital (BIH) Rawalpindi from March 2019 to May 2019 were included. Demographic details, history of blood transfusions and history of HD from any other center in addition to BIH, were recorded. Anti-HCV was done by chemiluminescent assay. Chi-square was used to compare the categorical variables. Odds ratio (OR) and relative risk (RR) for the groups exposed to risk were calculated.

**Results:**

Of 96 patients, 40 (41.6%) were anti-HCV positive. Sixty–two (64.6%) had transfusion history. Thirty-one (50%) of these 62 were anti–HCV positive as compared to 9 (26.5%) of 34 with no history of transfusion (p=0.025); OR=2.78 (p=0.0278), RR=1.89 (p=0.0420). Among 66(68.7%) of 96 who had HD from other centres in addition to ours, 33(50%) were anti-HCV positive as compared to 7(23.3%) of 23 who had HD from BIH only (p=0.014); OR=3.29 (p=0.0167), RR=2.14 (p=0.0309).

**Conclusion:**

There was a high prevalence (41.6%) of anti-HCV in our HD patients and anti-HCV positivity had significant association with history of blood transfusion as well as with history of HD from multiple centres.

## INTRODUCTION

Patients of end-stage kidney disease (ESKD) on maintenance haemodialysis (HD) are prone to contracting hepatitis C viral (HCV) infection which is a significant cause of morbidity and mortality in patients on maintenance HD.^1^ Increased risk of nosocomial infection by HBV or HCV in patients on HD has been attributed to impaired cellular immunity, blood transfusions, exposure to infectious materials through the extracorporeal circulation, frequent hospitalizations and surgery.^2^-^4^

The prevalence of anti-HCV is known to be higher in patients of ESKD on HD as compared to general population. Various studies done around the world have shown wide-ranging results regarding the prevalence of anti-HCV in patients on maintenance HD. In northern Europe the prevalence is less than 5%, in southern Europe and the United States around 10% and in many countries of northern Africa, Asia and South America, up to 70%.^4^,^5^

Among the factors responsible for increased risk of hepatitis in patients of ESKD on HD, blood transfusion is the most well-known. Although many studies have shown association of blood transfusion with increased sero-prevalence of anti-HCV in patients on HD, some have also refuted this relationship.^6^ In addition to blood transfusion, HD from multiple centres has also been identified as risk factor for acquiring HCV infections. This practice was also identified in some of our patients. This prompted us to carry out a study to determine the frequency of anti-HCV in the patients on maintenance HD and its association with (a) history of blood transfusion and (b) the practice of HD from more than one center.

## METHODS

This descriptive, cross sectional study was carried out at the Bahria Town International Hospital (BIH) Rawalpindi, Pakistan (Ethical Committee Review Ref. No. 001/2019 on July 7, 2019) from March 2019 to May 2019 employing consecutive nonprobability sampling. All the patients undergoing maintenance HD at the nephrology unit of BIH during the period of study were included. Patients of acute kidney injury requiring HD were excluded. In addition to demographic details, information about history of blood transfusions and history of HD from any other center in addition to BIH were recorded. Anti-HCV of all the patients was done by chemiluminescent micro-particle immunoassay on Architect ci8-200 (Abbott) chemistry analyzer. Patients were grouped into:

a. Those with the history of blood transfusion.
b. Those with no history of blood transfusion.

Frequencies and percentages of patients positive for anti-HCV in both the groups were compared by means of chi-square test. Similarly, patients were categorized into:

a. Those who had been having HD exclusively from BIH
b. Those who had also utilized the services of HD from other centres in Rawalpindi or any other city.

Frequencies and percentages of patients positive for anti-HCV in both the groups were compared by means of chi-square test. A *p*-Value of <0.05 was considered significant.Associations of anti-HCV prevalence with of blood transfusion and the practice of HD from more than one center were also assessed by calculating the odds ratio (OR) and relative risk (RR) along with their respective confidence intervals (CI) and *p*-values. A *p*-value of < 0.05 was considered significant.

## RESULTS

A total of 96 patients were included in the study. Male to female ratio was 1.8:1 with 62 (64.6%) males 34 (35.4%) females. Patients’ ages ranged from 17 to 81 years. Median age was 50 years while mean age was 47.84 (SD±14.75). Forty (41.6%) patients were anti-HCV positive. Distribution of patients into different age groups and the frequencies of anti-HCV positivity in each group are shown in Table-I.

**Table-I:** Frequency of anti-HCV positive and negative patients of ESKD on HD.

Age groups	Anti HCV positive	Anti HCV negative
Adolescents, <18 (n=2)	0 (0%)	2 (100%)
Young adults, 19-40 (n=30)	16 (53.3%)	14 (46.6%)
Middle age, 41-65(n=50)	19 (38%)	31 (62%)
Elderly, >65 (n=14)	5 (35.7%)	9 (64.3%)

Total	40 (41.6%)	56 (58.4%)

***Abbreviations:*** ESKD: End stage kidney disease, HCV: Hepatitis C Virus, HD: Haemodialysis.

Comparison of anti-HCV positive and anti-HCV negative ESKD patients on maintenance HD, on the basis of gender frequency, presence or absence of transfusion history and presence or absence of the history of HD from other center(s) in addition to BIH are shown in Table-II.

**Table-II:** Comparison of different groups of anti HCV positive and negative ESKD patients on maintenance HD (N=96).

	Anti HCV positive	Anti HCV negative	*Chi Square*	*p*-Value (significant if <0.05)
Male (n=62)	23 (37.1%)	39 (62.9%)	*X^2^=1.50*	*p=0.220*
Female (n=34)	17 (50%)	17 (50%)		
Patients with history of blood transfusion (n=62)	31 (50%)	31 (50%)	*X^2^=5.0*	*p=0.025*
Patients without blood transfusion history (n=34)	9 (26.5%)	25 (73.5%)		
Patients with history of HD from BIH only (n=30)	7 (23.3%)	23 (76.6%)	*X^2^=6.03*	*p=0.014*
Patients with history of HD from other centres in addition to BIH. (n=66)	33 (50%)	33 (50%)		

***Abbreviations:*** BIH: Bahria Town International Hospital, HCV: Hepatitis C virus, HD: Haemodialysis, ESKD: End stage kidney disease.

In patients with history of blood transfusion OR of being anti-HCV positive was 2.78 with 95% CI=1.1180-6.9019 and *p-Value=* *0.0278*, which was significant. This OR corresponded to the RR of 1.89 with 95% CI=1.0232-3.4870 and *p-Value=* *0.0420*, which was again significant.

In patients with history of HD from one or more other centres in addition BIH, OR of being anti-HCV positive was 3.29 with 95%CI=1.2406-8.7022 and *p-Value=0.0167* (which was significant) and the corresponding RR was 2.14 with 95% CI=1.0726-4.2811 and *p=0.0309* (significant).

## DISCUSSION

Presence of anti-HCV in a patient is interpreted as past exposure to HCV at some point in time irrespective of the current viraemic status of the patient. Because of its strong association with HCV hepatitis it has been used as an epidemiological marker for HCV. According to WHO estimates, more than 71 million people were living with chronic HCV infection worldwide in 2015 and an estimated 1.75 million new HCV infections occurred during the same year.^7^ A study based on meta-analysis has reported overall prevalence of HCV in Pakistan as 6.2%.^8^ Another very large study from Rawalpindi on blood donors has reported the sero-prevalence of anti-HCV as 2.6%.^9^

Prevalence of anti-HCV is known to be more in patient undergoing maintenance HD than in the general population. We found 41.6% of our patients on HD to be positive for anti-HCV. Highest percentage (53.3%) of anti-HCV positivity was seen in young adults (19-40 age group). Very few studies are available from Pakistan on the topic of anti–HCV sero-prevalence in HD patients. A study done at a hospital in Lahore reported the prevalence of anti-HCV in patients on HD as 68%.^10^ Literature from other countries has shown extreme variation in the prevalence of anti-HCV in HD patients. Studies from European, and North American countries such as UK, Germany and US have low prevalence i.e. 4%, 6% and 7.3% respectively.^6^,^11^,^12^ In Russia, Saudi Arabia, Libya and Sudan the prevalence is 14%, 20.5% and 23.7% respectively.^6^,^12^ Prevalence of anti-HCV in excess of 40% has been reported from, Syria, Iran, Tunis, Senegal, Moldavia, Bosnia, Cuba, Brazil and Peru.^3^,^12^

In our study, 31 (50%) of 62 patients with history of blood transfusion were positive for anti-HCV, showing strong association between the two. Both the OR and RR of being anti-HCV positive were also significantly high in patients with blood transfusion history. However, a significant contribution to the magnitude of sero-prevalence can be expected from other concurrent nosocomial risk factors, resulting in overestimation of association. This notion stems from the fact that the sero-prevalence of anti-HCV has reduced in blood donors in our region and application of more stringent methods of blood donors’ screening have decreased the chances of spread of hepatitis to the recipients over the past few years.^9^

Most of the studies have shown that the history of blood transfusions is associated with an increased prevalence of anti-HCV in dialysis patients. A few reports from Saudi Arabia, Iran and Syria however, did not identify blood transfusion as an independent risk factor in HCV spread among HD patients.^13^-^15^

Some patients of ESKD receive HD treatment from more than one center due to various reasons. This practice has been reported in different studies to constitute a risk factor for acquisition of HCV.^3^ Sixty-six (68.7%) of 96 patients included in our study admitted to have had HD from at least one other center in addition to ours. Prevalence of anti-HCV in these patients was more than twice as compared to those who had HD exclusively from our hospital. Our study validates the findings of earlier studies regarding the association of anti-HCV positivity with the practice of HD from multiple centres. With less than 300 dialysis centres in the country and more than 20,000 new patients of ESKD every year, these facilities are grossly overburdened.^16^,^17^ Overworked staff of the dialysis centres, especially those of the public hospitals, which handle a big proportion of patients’ burden, are liable to commit lapses in observing standard precautions, resulting in spread of HCV to previously unexposed patients.

## CONCLUSION

There was a high prevalence (41.6%) of anti-HCV in our HD patients and anti-HCV positivity had significant association with history of blood transfusion as well as with history of HD from multiple centres.

## RECOMMENDATIONS

The situation demands strict implementation of standard precautions and implementation of policies framed in the light of internationally recognized guidelines such as those of KDIGO (Kidney Disease: Improving Global Outcomes).

### Authors’ Contributions:

**SQA** conceived, designed, statistical analysis.

**UA and SM** data collection, manuscript writing.

**MA** Data collection, manuscript editing, review and approval of manuscript.
